# Quantitative Profiling of Phenolic Constituents in *Hypericum perforatum* L. via HPLC–PDA and HPLC–ECD: A Chemometric Approach

**DOI:** 10.3390/molecules30193854

**Published:** 2025-09-23

**Authors:** Andrin Tahiri, Zamir Damani, Dritan Topi

**Affiliations:** 1 Medical Service of Toxicology and Addictology, Mother Teresa’ University Hospital Center, 1005 Tirana, Albania; andrin.tahiri@qsut.gov.al; 2 Department of Diagnostics, Faculty of Technical Medical Sciences, University of Medicine of Tirana, Kongresi I Manastirit Street, No. 133, 1005 Tirana, Albania; zamir.damani@umed.edu.al; 3 Department of Chemistry, Faculty of Natural Sciences, University of Tirana, Blvd Zog I, 1001 Tirana, Albania

**Keywords:** *Hypericum perforatum* L., St. John’s wort, liquid chromatography, phenolic compounds, hyperforin, chemoinformatics, Albania

## Abstract

(1) Background: Medicinal plants are widely used in folk medicine. *Hypericum perforatum* L. (St. John’s wort) is a medicinal plant that is used domestically and exported to other countries. This study addresses the need to develop methods for determining the composition and content of St. John’s wort to determine its biological activity. (2) Methods: High-performance liquid chromatography (HPLC) equipped with an Electrochemical Detector (ECD) and a Photodiode Array Detector (PDA) was employed to identify and quantify major phenolic compounds—gallic acid, catechin, epicatechin, hyperoside, quercetin, and hyperforin—in extracted and lyophilized St. John’s wort flower; stem; and leaf samples. Key analytes exhibited linear responses across both detection systems, within a quantification range of 0.5–10 µg/mL. (3) Results: The PDA method, validated according to ICH Q2(R1) guidelines, demonstrated specificity, linearity, precision, and accuracy, with limits of detection (LOD) ranging from 0.24 to 0.61 µg/mL and limits of quantification (LOQ) between 0.26 and 0.62 µg/mL. PDA effectively identified gallic acid, epicatechin, hyperoside, quercetin, and hyperforin, although catechin was not detected. ECD yielded comparable compound levels across the samples. (4) Conclusions: The novelty of this study lies in identifying the influence of climatic factors associated with the altitude at which St. John’s wort is grown on the content and ratio of biologically active components. Overall, the chemometric approach demonstrates the utility of raw chromatographic data in distinguishing samples by plant part and geographic origin; even when traditional compound-based comparisons may be limited.

## 1. Introduction

Medicinal plants, as part of folk medicinal remedies, have been used for a long time, and evidence of medicinal plant applications by the peoples of the Balkans is well documented from antiquity [1]. Southeast Europe is among Europe’s most important export regions for medicinal and aromatic plants. Approximately 8% of the total global Medicinal and Aromatic Plants (MAPs) export volume originated from this region during the 1990s [2]. Compared with the country’s size, Albania’s floral diversity is very high and distinguished, with over 3630 plant species belonging to 960 genera and 175 families [3,4]. The country is also rich in medicinal and aromatic plants, with approximately 250 species. In terms of quantity, Albania is a leading exporter in Southeast Europeand is identified as one of the world’s major suppliers [5,6]. St. John’s wort is a yellow-flowering, perennial herb native to Europe, West Asia, and North Africa [7,8,9]. This plant belongs to the genus Hypericum in the Hypericaceae family [10]. It comprises approximately 450 species, ranging from annual or perennial herbs to shrubs and trees, in all tropical and temperate regions of the world [11,12]. *Hypericum perforatum* L. (St. John’s wort) is a widely studied medicinal plant known for its antidepressant, anti-inflammatory, and antimicrobial properties. Its phytochemical profile includes a wide range of bioactive compounds such as hypericin, hyperforin, flavonoids, and phenolic acids. Despite extensive research, the quantification and differentiation of these compounds remain challenging due to variability in plant origin, extraction methods, and analytical techniques. In Albania, *H. perforatum* is present across the country between 300 and 1500 m above sea level. In folk medicine, this plant is used as an antidepressant, to aid in wound healing, and to treat liver disorders and rheumatism [5]. The ancient Greeks were aware of the medicinal properties of this genus. St. John’s wort was recognized by Hippocrates, Pliny, and Dioscorides [13]. Its chemical composition and pharmacological properties make *H. perforatum* one of the most widely studied medicinal plants. *H. perforatum* extracts have been partially chemically characterized, and more than 150 compounds have been identified [14]. The largest group belongs to the flavonol glycosides (up to 4%) [15]. The major flavonoids are rutin, hyperoside, isoquercitrin, quercitrin, miquelianin, and quercetin [16]. Crude extracts of the flowers and leaves contain the naphthodianthrones hypericin (0.03–0.3%) and pseudohypericin at 2–10 times the level of hypericin. Hyperforin is identified as the most important compound in the phloroglucinol group, exhibiting antidepressant activity through a novel mechanism of action, as well as antibiotic activity against Gram-positive bacteria and antitumoral activity in vivo [17]. Studies on the chemical composition of the *H. perforatum* essential oils indicate that oxygenated sesquiterpenes (41.4%) and caryophyllene oxide (31.0%) are the main constituents [18].

St. John’s wort is a nutraceutical, phytopharmaceutical, and cosmetic ingredient. The Hyperici herbal extract is an active drug [19]. Hypericum extracts are used to control anxiety, as an anticonvulsive, and in pain management, being used topically for wound treatment and in dental practice. Additionally, studies in laboratory animals have evaluated the effectiveness of *H. perforatum* preparations in treating cancer, inflammation-related disorders, bacterial and viral diseases, and as an antioxidant and neuroprotective agent [20]. The phytochemicals that have attracted the most attention for their phytopharmaceutical activities include hypericin, pseudohypericin, rutin, quercetin, quercitrin, miquelianin, amentoflavone, hyperoside, hyperforin, and adhyperforin. The phytochemical composition of St. John’s wort varies depending on cultivar, climate, horticultural practices, growing conditions, stage of maturity, method and timing of harvest, and extraction procedures.

High-performance liquid chromatography (HPLC) is the most commonly used method for analyzing *H. perforatum* constituents. However, many studies rely solely on UV or PDA detection, which may lack sensitivity or selectivity for certain compounds, especially those with electroactive properties. Electrochemical detection (ECD), although less frequently applied, offers enhanced sensitivity for phenolic compounds but is underutilized in phytochemical profiling. Moreover, most existing studies focus on isolated compound quantification rather than leveraging raw chromatographic data for chemometric discrimination.

Therefore, this study aims to develop and validate a dual-detection HPLC method (PDA and ECD) for the comprehensive profiling of phenolic constituents in *H. perforatum* extracts collected from diverse Albanian regions. By integrating chemometric tools such as Principal Component Analysis (PCA) and Cluster Analysis (CA), we explore the potential of raw chromatographic data to distinguish samples based on geographic origin and plant part. This approach addresses the limitations of traditional compound-based comparisons and contributes novel insights into the influence of environmental factors on phytochemical variability.

## 2. Results and Discussions

This study investigated the variation in *H. perforatum* extracts collected from different Albanian regions, focusing on how altitude and temperature influence their chemical profiles (Table 1). A comprehensive chromatographic analysis was conducted and compared with the existing literature on Hypericum species. Dresler et al. (2018) [21] developed both isocratic (VWD) and gradient (PDA) HPLC methods for quantifying hyperforin, hypericin, chlorogenic acid, quercitrin, quercetin, rutin, and hyperoside, including epicatechin. Our method demonstrated a shorter runtime, with six standards eluting within 20 min (PDA) and 27.5 min (ECD). Notably, ECD achieved faster elution and better resolution, although hyperoside and rutin were not efficiently separated (R < 0.75). The significantly faster elution observed in our method can be attributed to several key optimizations. First, we employed a more efficient solvent system that enhanced analyte mobility and reduced retention time. Second, the flow rate and pressure parameters were carefully adjusted to accelerate the chromatographic process without compromising resolution. Third, the selection of a column with tailored physicochemical properties allowed for improved interaction with the target compounds, facilitating faster separation. These combined improvements reflect a deliberate methodological refinement aimed at increasing analytical throughput while maintaining accuracy and reproducibility.

In contrast to previous methods, our PDA and ECD approaches achieved clear separation of all six standards, with resolution values ranging from 0.93 to 4.88 (PDA) and 1.57 to 9.15 (ECD). The calculated LOD and LOQ value ranges for PDA were 0.24–0.61 µg/mL and 0.26–0.62 µg/mL, respectively, aligning well with published data. Seyis et al. (2020) [22] used an RP-18 column (250 mm × 4.0 mm, 5 µm) with PDA detection to separate flavonoids and phenolic acids.

**Figure 1:**
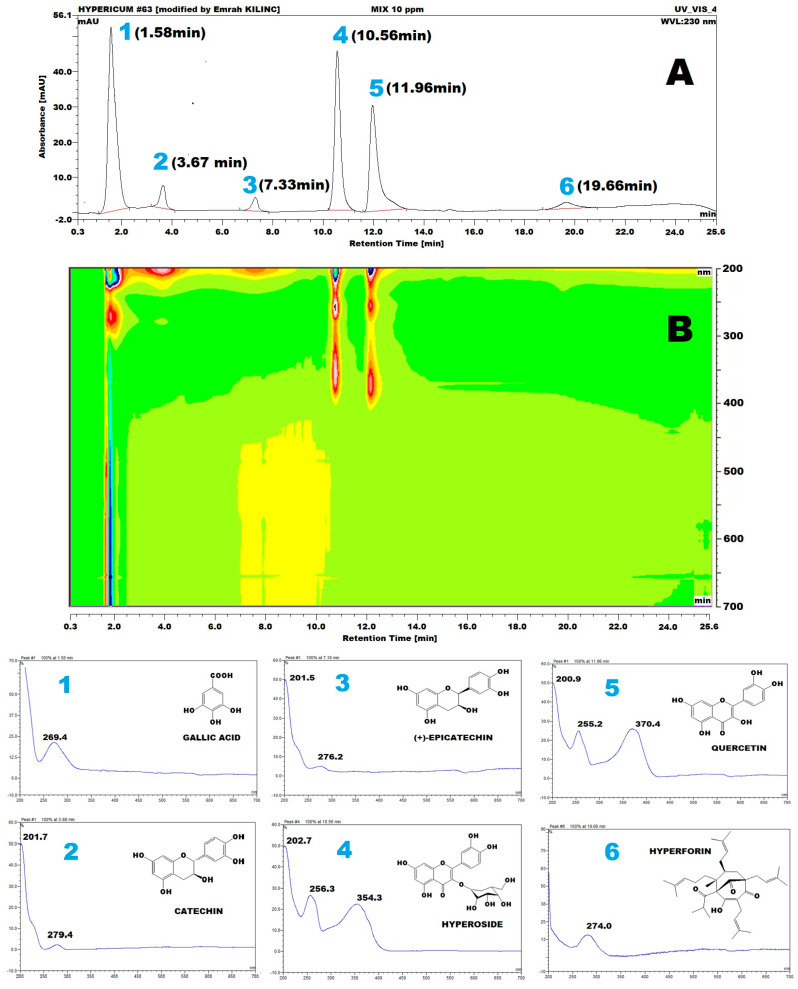
Chromatograms at 230 nm conventional (**A**) and 200–700 nm range 3D isoplot chromatograms (**B**) and specific UV-Vis spectra (**C**) of 10 µg/mL gallic acid (**1**), catechin (**2**), epicatechin (**3**), hyperoside (**4**), quercetin (**5**), and hyperforin (**6**). The mobile phase combination of aqueous 0.01 M phosphoric acid and acetonitrile was used with a C18 250 × 4.6 mm, 5 µm, 100 Ǻ analytical column at a 0.25 mL/min flow rate.

To clarify, the LOD and LOQ values reported in the Abstract (LOD: 0.24–0.61 µg/mL; LOQ: 0.26–0.62 µg/mL) represent the range calculated for all analytes using the PDA detector. These values were derived from the standard deviation of the response and the slope of the calibration curve, following ICH Q2 (R1) guidelines. Although LOQ is theoretically expected to be about three times higher than LOD, compounds with steep calibration slopes and low signal variability may yield numerically similar values. The LOD of 2.5 ng/mL for hyperforin refers to a previously published study by Rückert et al. [23]. As all the antioxidant standards have electroactive functional groups (Figure 1), ECD can monitor any possible standard in the extracts.

Focusing on *Hypericum perforatum* extracts of Northern Türkiye—specifically targeting hypericin, pseudohypericin, hyperforin, the chlorogenic, neochlorogenic, caffeic, and 2,4-dihydroxybenzoic acids, isoquercitrin, quercitrin, avicularin, hyperoside, rutin, biapigenin, (+)-catechin, and (−)-epicatechin—phytochemical content information was obtained from an altitude range of 391–2210 m. The results of the analysis indicate that plants at higher locations produce the highest phytochemical contents. This finding is also in harmony with our results, as phenolic extracts from the site (Pogradec) at an altitude of 700–1000 m similarly had the highest total polyphenol content, at 192.55 and 201.23 µg/mL, respectively, in both leaf and flower phenolic extracts. Seyis and colleagues (2020) [24] calculated LOD and LOQ values in the ranges of 0.019–1.022 and 0.19–10.22 mAU, respectively, which resulted in higher values compared with LOD (0.0012–0.0312 mAU) and LOQ (0.014–0.104 mAU) in the proposed method, indicating that our method is approximately 10 to 100 times more sensitive, depending on the compound of interest. In their published article, Yao and colleagues (2019) [25] employed an isocratic method for HPLC-PDA analysis of hypericin at 588 nm using an analytical column of Eclipse XDB-C8 at 60 °C with dimensions of 150 mm × 4.6 mm and 5 μm particle size. The flow rate and injection volumes were 1.0 mL/min and 20 µL, respectively. The mobile phase comprised 0.03 mol/L KH_2_PO_4_ (adjusted to pH 7.0 with 0.5 mol/L K_2_HPO_4_) and methanol (30:70, *v*/*v*). Their method details and parameters are barely different from our parameters and conditions. They demonstrated that the bioactive compound biosynthesis and antioxidant capacity of *Hypericum perforatum* extracts were induced via exposure to a lower temperature of 15 °C compared with 22 °C or 30 °C. This finding aligns with our results, which also demonstrate that total antioxidant ingredients are higher at higher altitudes (lower temperatures). Altitudinal changes in secondary metabolite contents of *Hypericum* sp. are also investigated in another study by Cirak and colleagues (2017) [26], where they identified hypericin, pseudohypericin, hyperforin, adhyperforin, chlorogenic acid, neochlorogenic acid, caffeic acid, 2,4-dihydroxybenzoic acid, 13,II8-biapigenin, hyperoside, isoquercitrin, quercitrin, quercetin, avicularin, rutin, (+)-catechin, and (−)-epicatechin in aerial parts of the plants growing at different altitudes in Türkiye. They developed a gradient method using a C18 column (150 mm × 3.0 mm, 3.5 µm) at 25 °C, with mobile phases A (0.3% H_3_PO_4_ in water) and B (0.3% H_3_PO_4_ in acetonitrile), with a flow rate of 0.6 mL/min, using a 30 min running time, which is approximately 50% longer than our method. Although the study presents a developed method, no validation data is provided. At the same time, all compounds were intensively determined in plants at higher altitudes. The hypothesis they proposed to explain this phenomenon is that alterations in the levels of these compounds may play a critical role in enabling plants to overcome the abiotic stress of lower temperatures and higher ultraviolet (UV-B) radiation, which is expected to be elevated at higher altitudes. Temerdashev and colleagues (2020) [27] developed two different gradient HPLC-PDA methods: one method for the determination of protocatechuic acid, neochlorogenic acid, chlorogenic acid, (−)-epicatechin, rutin, hyperoside, isoquercitrin, quercitrin, quercetin, and 13, II8-biapigenin, while the other method is used for urohyperforin, hyperforin, adhyperforin, pseudohypericin, and hypericin. The first method employs a run time of 30 min, while the latter has a 12 min run time. They employed a C18 column (250 mm × 4.6 mm, 5 μm particle size) at 40 °C, with mobile phases A (acetonitrile and 0.1% formic acid) and B (H_2_O). The developed methods, which are different from those in our study, studied the stability of hyperforin in *Hypericum perforatum* L. extracts of plant materials collected from various locations in Russia. Zorzetto and colleagues (2015) [23] performed a phytochemical analysis and in vitro biological activity study of three *Hypericum* sp. species from the Canary Islands. They developed a gradient HPLC-PDA method for determining the concentrations of hypericin, hyperforin, chlorogenic acid, rutin, hyperoside, isoquercitrin, quercitrin, and quercetin. The analytical column was a Phenomenex C18 (250 mm × 4.6 mm, 5 μm), with mobile phase A (ultrapure water, 0.02% H_3_PO_4_ pH 2.7), mobile phase B (AcCN/MeOH, 90:10, *v*/*v*), and mobile phase C (ethyl acetate/mix of eluent A/B 10:90 *v*/*v*), employing a flow rate of 1.0 mL/min, absorbance at 210 nm, and a running time 130 min. The optimized parameters of the method are mainly unchanged from those in the current study, and the running time is significantly longer. A partial validation process was performed.

**Figure 2:**
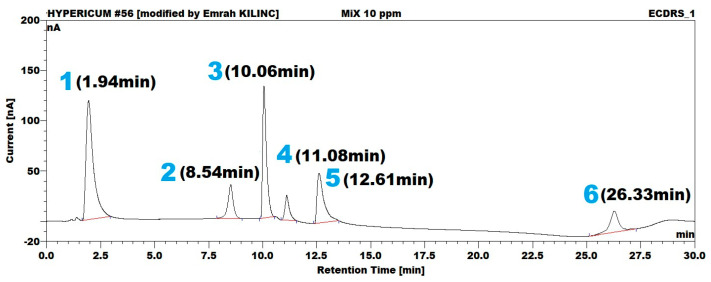
Representative ECD chromatogram of a 10 µg/mL standard mixture containing gallic acid (**1**), catechin (**2**), epicatechin (**3**), hyperoside (**4**), quercetin (**5**), and hyperforin (**6**).The mobile phase combination of aqueous 0.01 M phosphoric acid and acetonitrile was used with a C18 250×4.6 mm, 5 µm, 100 Ǻ analytical column at a 0.25 mL/min flow rate and +750 mV (vs. Ag/AgCl) potential.

Similar analytical column and mobile phase conditions were employed for ECD detection. In contrast, the integrated column department was maintained at 25 °C, and the ECD operating potential was set to +750 mV (vs. Ag/AgCl) using a glassy carbon electrode, with a data collection rate of 10 Hz. A representative HPLC-ECD chromatogram (Figure 2)shows that the corresponding retention times were 1.94, 8.54, 10.06, 11.08, 12.61, and 26.33 min for gallic acid (1), catechin (2), epicatechin (3), hyperoside (4), quercetin (5), and hyperforin (6), respectively.

**Figure 3:**
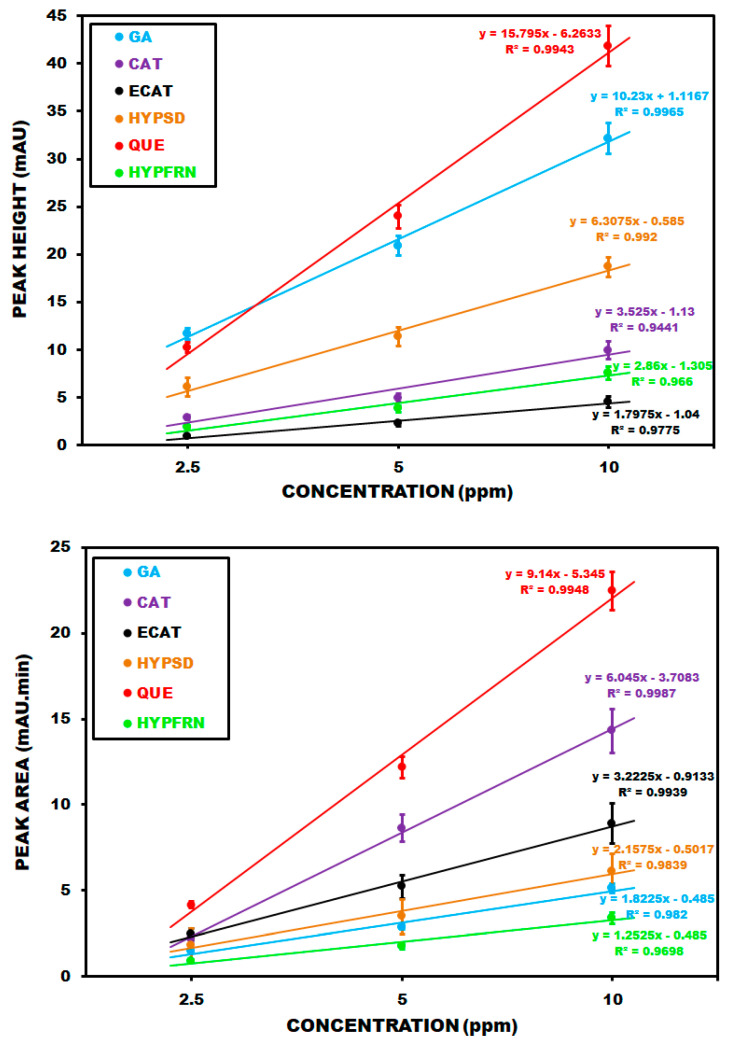
PDA calibration plots and related regression equations of the standards based on peak heights and areas.

Corresponding calibration plots for PDA detection based on peak height (A) and peak area (B) are summarized in Figure 3. The regression equations for gallic acid (GA), catechin (CAT), epicatechin (ECAT), hyperoside (HYPSD), quercetin (QUE) and hyperforin (HYPFRN) are y = 10.23x + 1.1167 (R^2^ = 0.9965), y = 3.525x − 1.13 (R^2^ = 0.9441), y = 1.7975x − 1.04 (R^2^ = 0.9775), y = 6.3075x−0.585 (R^2^ = 0.9920), y = 15.795x − 6.2633 (R^2^ = 0.9943), and y = 2.86x − 1.305 (R^2^ = 0.9660), respectively, based on peak heights for a concentration range of 1–10 ppm. Regarding peak areas, the regression equations for the same compounds and concentration range arey = 1.8225x − 0.485 (R^2^ = 0.982), y = 6.045x − 3.7083 (R^2^ = 0.9987), y = 3.2225x − 0.9133 (R^2^ = 0.9939), y = 2.1575x − 0.5017 (R^2^ = 0.9839), y = 9.14x − 5.345 (R^2^ = 0.9948), and y = 1.2525x − 0.485 (R^2^ = 0.9698).

The most important antioxidant phytochemicals of *Hypericum perforatum* are hyperforin, hypericin, and hyperoside. There are very few studies in the literature that employ the HPLC-ECD method. Rückert and colleagues (2004) [23] developed an isocratic method using a C18 column (100 × 4.6 mm, 5 µm). Hyperforin was detected in Hypericum perforatum-containing herbal medicinal products at +1.1 V (vs. Ag/AgCl), with linearity achieved over the 0.054–5.4 µg/mL concentration range. LOD and LOQ were calculated to be 2.5 and 54 ng/mL, respectively. Partial validation was conducted to assess the accuracy, precision, limit of detection (LOD), and limit of quantification (LOQ) parameters. Rückert and colleagues focused solely on hyperforin, excluding the other flavonoids, and limited their study to herbal medicinal products rather than extracts from various regions or locations. Hyperforin mainly accumulates in pistils and fruits, which probably serves as a defensive compound [17].

**Figure 4:**
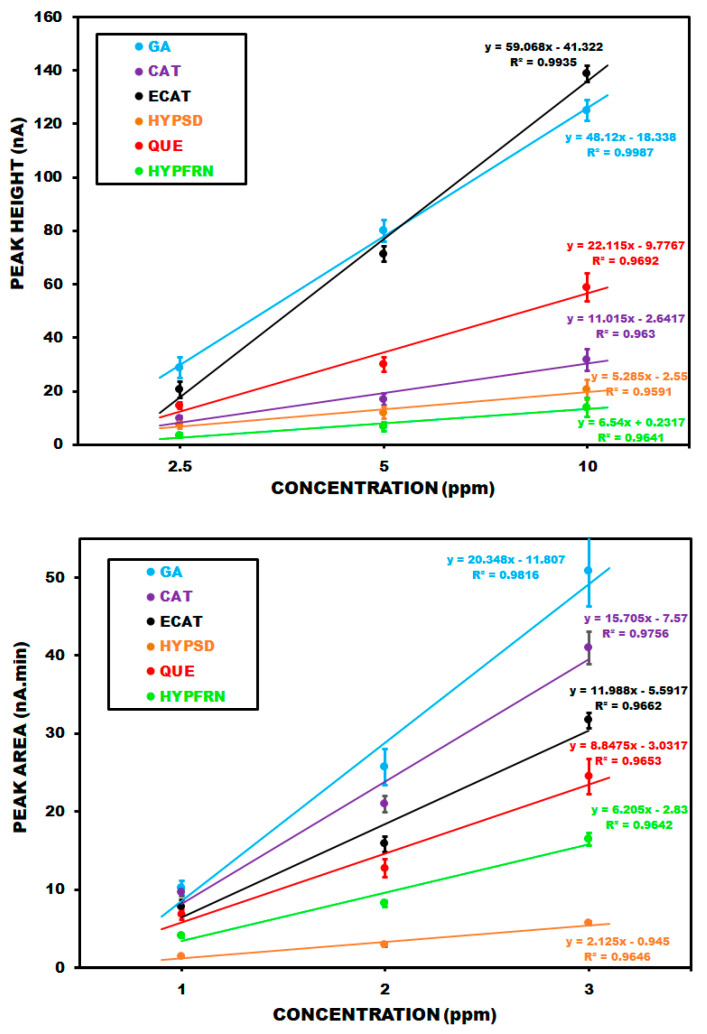
ECD calibration plots and related regression equations of the standards based on peak heights and areas.

**Figure 5:**
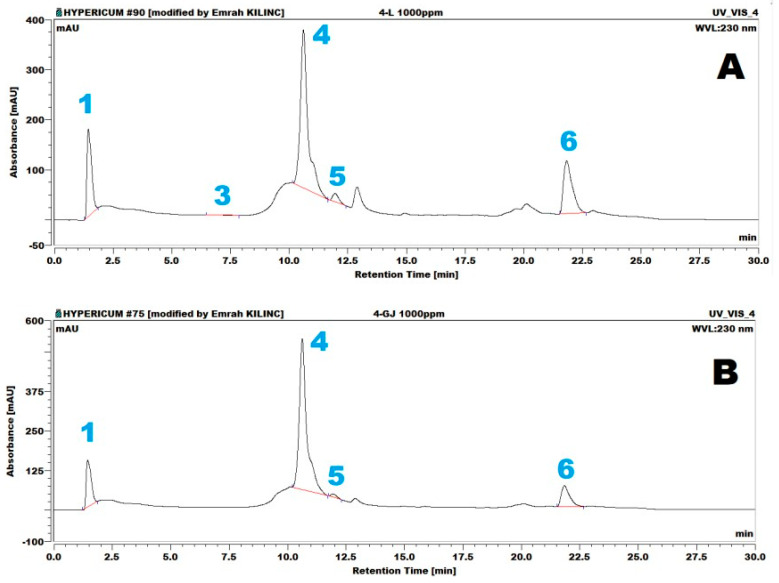
Representative PDA chromatograms of the 4-L/Fl (**A**) and 4-GJ/SL (**B**) samples were recorded at 230 nm.

HPLC-ECD analysis employed electrochemical calibration plots based on peak height (A) and peak area (B), as summarized in Figure 4. The regression equations for gallic acid (GA), catechin (CAT), epicatechin (ECAT), hyperoside (HYPSD), quercetin (QUE) and hyperforin (HYPFRN) arey = 48.12x − 18.338 (R^2^ = 0.9987), y = 11.015x − 2.6417 (R^2^ = 0.9630), y = 59.068x − 41.322 (R^2^ = 0.9935), y = 5.285x − 2.55 (R^2^ = 0.9591), y = 22.795x − 7.7767 (R^2^ = 0.9692), and y = 6.54x + 0.2317 (R^2^ = 0.9641), respectively, based on peak heights for a concentration range of 1–10ppm. Regarding peak areas, the regression plots for the same compounds and similar concentration range arey = 1.8225x − 0.485 (R^2^ = 0.982), y = 6.045x − 3.7083 (R^2^ = 0.9987), y = 3.2225x − 0.9133 (R^2^ = 0.9939), y = 2.1575x − 0.5017 (R^2^ = 0.9839), y = 9.14x−5.345 (R^2^ = 0.9948), and y = 1.2525x − 0.485 (R^2^ = 0.9698), respectively. Representative HPLC-PDA chromatograms of the samples are presented in Figure 5. These chromatograms belong to the samples from the Gjirokastraarea, composed of flower (Fl) and stem and leaf (SL) sections of the plant. As shown in the figure, gallic acid (1), (+)-epicatechin (3), hyperoside (4), quercetin (5), and hyperforin (6) were identified in the flowers. The same compounds, except for (+)-epicatechin (3), were identified in the leaf material.

The results of the entire HPLC analysis (PDA and ECD) (Table 1) indicate that catechin was the only flavonoid absent in the whole plant extracts, as detected using both detectors. Regarding PDA, hyperoside and hyperforin were the major flavonoids, determined at 96.12 and 142.64 ppm in the 5-(SL) and 3-Fl extracts, respectively. In the case of the ECD detection method, hyperoside and hyperforin were the major flavonoids, quantified as 101.48 and 128.40 ppm in 5-(SL) and 3-(Fl) methanolic extracts, respectively.

**Figure 6:**
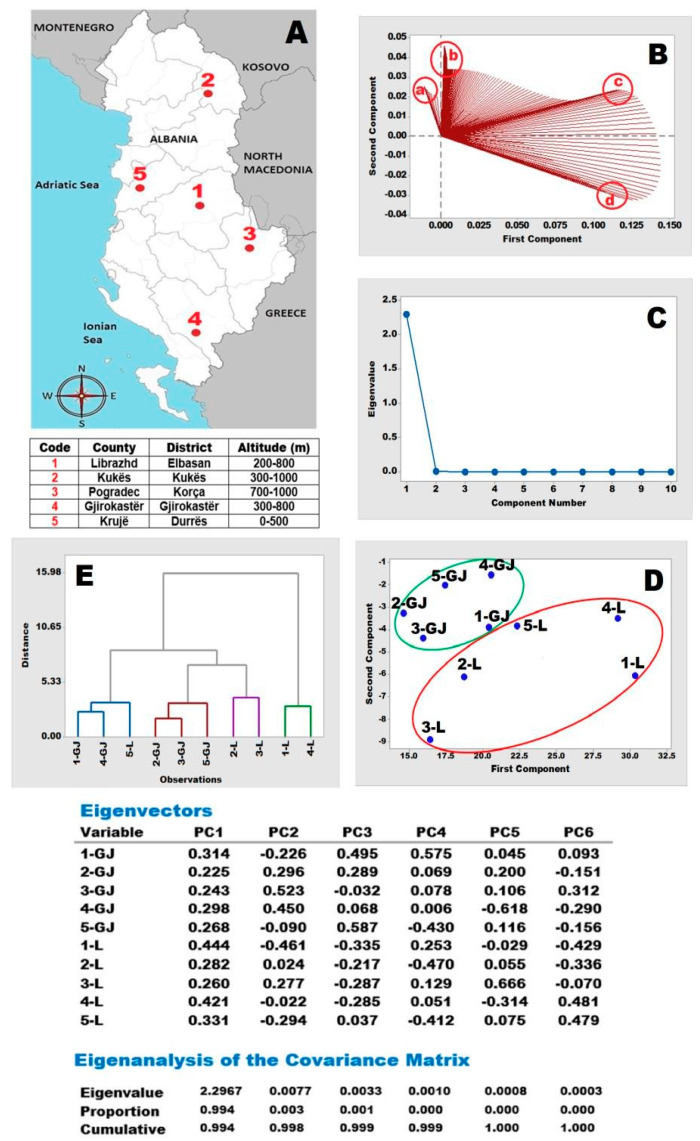
Chemoinformatics based on PDA raw data of the extracts from related locations and altitudes (**A**) are summarized inthe loading plot (**B**), scree plot (**C**), PCA score plot (**D**), and CA dendrogram (**E**) together with the Eigenvectors table. Abbreviations: Gj:SL (stem and leaf); L:Fl (flower).

The total flavonoid content produced through HPLC-PDA in the methanolic extracts revealed that sample 3-(Fl) had the highest concentration at 201.23 ppm, followed by 3-(SL), 5-(Fl), 2-(Fl), and 5-(SL),correspondingto192.55, 182.43, 148.20, and 142.78 ppm, respectively. Similarly, the HPLC-ECD results showed that sample 5-(SL) had the highest flavonoid content at 187.13, followed by 5-(Fl), 3-(Fl), 3-(SL), 2-(Fl), and 5-(SL), with total flavonoid levels of 185.52, 182.53, 147.07, and 146.54 ppm, respectively. These findings emphasize the variation in flavonoid concentration across different plant parts and geographic origins, as detected using both PDA and ECD methods. The highest concentrations of gallic acid, hyperoside, quercetin, and hyperforin were detected in samples 4-(SL), 5-(Fl), 5-(SL), 3-(Fl), and 3-(Fl), regardless of the detector used (PDA or ECD). The results of chemometric discrimination are based on raw PDA (Figure 6), with the locations of plant materials identified on the map (A) and accompanied by location and altitude information. The chromatograms in Figure 5 show the results of extracts from the flower (Fl) and stem and leaf (SL) samples collected in the Gjirokastraarea.

The sample locations were selected based on their exposure to the climate to determine the influence of climate on hypericin composition. Three sample locations (Kruja, Gjirokastra, and Librazhdi) are under the influence of the Mediterranean climate, characterized by mild winters and warm, humid summers, compared with the two other locations in the Pogradeci and Kukësareas, which are under a subalpine to alpine climate [28].

The chemometric analysis was based on raw chromatographic data obtained from PDA and ECD detectors, specifically peak areas and heights of the five detected compounds: gallic acid, epicatechin, hyperoside, quercetin, and hyperforin. Although catechin was included in the standard mix, it was not detected in any of the samples; therefore, it was excluded from statistical analysis. A Principal Component Analysis (PCA) was performed using the normalized dataset, and six principal components were extracted. The presence of six PCs reflects the dimensionality of the raw data matrix, which includes multiple wavelength channels and retention time intervals, not just compound counts. The PCA results from PDA data showed clear separation of the samples based on plant part, with the flower samples (Fl) clustering distinctly from the stem and leaf samples (SL). This suggests that tissue-specific phytochemical profiles significantly influence the variance captured by the principal components. In contrast, the ECD-based PCA grouped samples from localities 3 and 4 together, and those from 2 and 5 together, despite differing altitudes and climates. This pattern may be attributed to shared electroactive compound profiles or similar redox behavior under the applied detection potential. The loading plot (B) displays the specific time ranges as four identified orientations on the plane marked as *a, b, c*, and *d*. These correspond to PDA data fractions recorded at 1.22–1.32 min, 5.10–5.17 min, 8.16–8.19 min, and 18.06–18.08 min, respectively.

At this time, the distribution of PDA data exhibited noticeable variations from the general trend; thus, the data acquired was significant and helpful for chemoinformatic tools such as PCA. The scree plot (C) displays the number of principal components required to explain the total variance present in the recorded PDA data. Using principal components PC1, PC2, and PC3, 99.4%, 99.8%, and 99.9% of the total variance becomes explainable, respectively. The PCA results are graphed in the score plot (D), where it is interesting to note that very similar distributions are observed on the plane for both the leaf and flower samples. Although only five compounds were detected, the raw data matrix includes multiple variables per compound (e.g., absorbance at different wavelengths, retention time slices), resulting in a higher number of principal components. The six PCs reflect the complexity of the raw data rather than the number of compounds alone. Regarding the flower material, the 1-(Fl) and 4-(Fl) samples appear to be distinct from the remaining ones. These samples originate from Librazhdi and Gjirokastra counties, which have almost identical altitude ranges, and this may be associated with their discrimination. Changes in orientations of 1-(Fl) and 4-(Fl) on the plane may also be related to the presence of other ingredients (e.g., polyphenols, flavonoids, other aromatics, and ions absorbing light in the recorded 300–700 nm range) in the methanolic extracts, making some contribution to the separation of the samples. Additionally, the 2-(Fl) and 3-(Fl) samples are also located closer to each other inaltitude, and this may be related to their ingredients and/or location altitudes, as they belong to Kukës and Pogradeci counties, respectively.

The grouping observed in the PDA-based PCA (flowers vs. stems/leaves) is consistent with tissue-specific phytochemical profiles. Regarding the ECD-based PCA, the grouping of localities 3 and 4 versus 2 and 5 may be influenced by shared electrochemical behavior of compounds under the applied potential, rather than altitude or climate alone; however, this interpretation requires further elaboration.

The influence of altitude on the antioxidant profile of *H. perforatum* was evident in our findings. The samples collected from higher elevations—specifically those above 700 m above sea level, such as Pogradeci and Kukës—exhibited significantly higher concentrations of total flavonoids and key antioxidant compounds, including hyperforin and hyperoside. This trend suggests that altitudinal thresholds around 700 m may mark a biologically relevant point where environmental stressors such as lower temperatures and increased UV-B radiation begin to stimulate the biosynthesis of secondary metabolites. These observations are consistent with previous studies [22,25], indicating increased phytochemical accumulation in *Hypericum* species cultivated at elevated altitudes.

Representative HPLC-ECD chromatograms of the samples are presented in Figure 6. As shown in Figure 6, gallic acid (1), (+)-epicatechin (3), hyperoside (4), quercetin (5), and hyperforin (6) were identified in both the flower (Fl) and leaf (SL) samples belonging to the Gjirokastra area.

**Figure 7:**
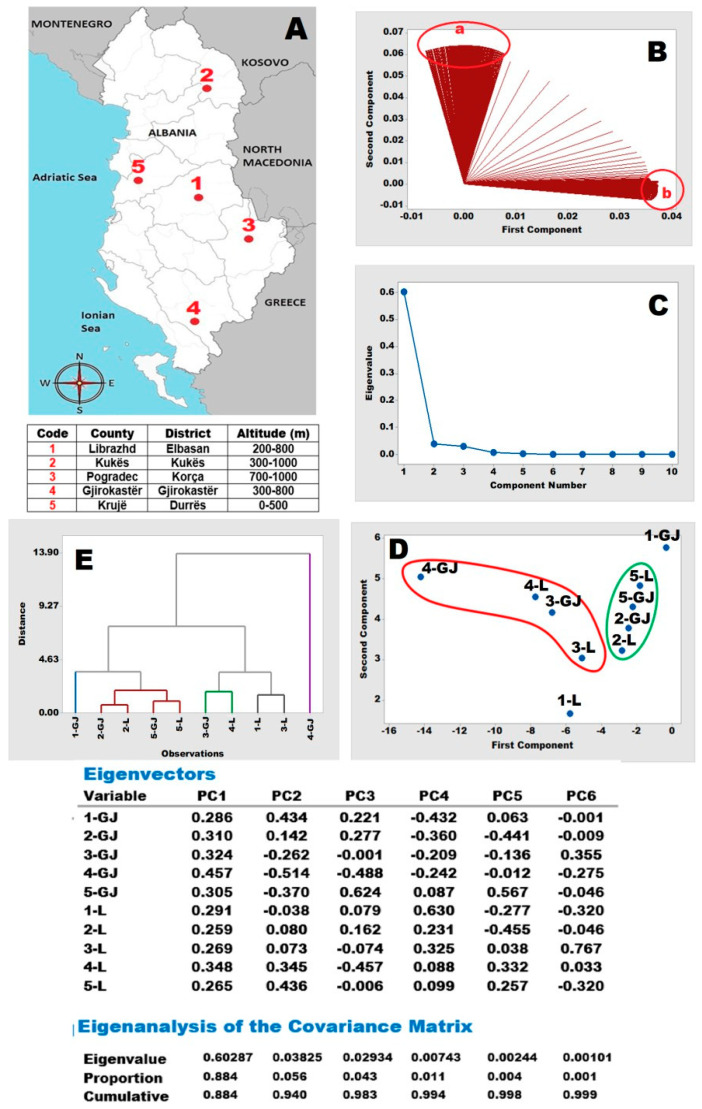
Chemoinformatics based on ECD raw data of the extracts from related locations and altitudes (**A**) are summarized inthe loading plot (**B**), scree plot (**C**), PCA score plot (**D**), and CA dendrogram (**E**) together with the Eigenvectors table.

The raw data on the phenolic compounds in the leafsamples present a very similar distribution across locations. The Cluster Analysis, as shown in the dendrogram (E), clearly distinguishes four main clusters, which are defined by Euclidean distances. These clusters are formed by 1-(SL), 4-(SL), 5-(Fl) (blue); 2-(SL), 3-(SL), 5-(SL) (red); 2-(Fl), 3-(Fl) (violet); and 1-(Fl), 4-(Fl) (green). In the PCA score plot (D), their locations on the plane are similarly related to the dendrogram (E). Figure 7 provides the outcomes of chemoinformatics based on raw HPLC-ECD data. The location information of the collected plant materials is summarized on the country map (A), where number codes 1–5 are used for Librazhdi (200–800 m), Kukës (300–1000 m), Pogradeci (700–1000 m), Gjirokastra (300–800 m), and Kruja (0–500 m), respectively.

Two specific identified orientations on the plane, marked as *a* and *b*, correspond to ECD data fractions recorded in time ranges of 5.40–6.30 min and 8.80–9.20 min, respectively. The pattern of ECD data acquired is significant and helpful for performing chemoinformatics. The scree plot (C) displays the number of principal components (PCs) necessary for expressing total variance present in the recorded ECD data, depending on principal components 1, 2, 3, and 4. 88.4%, 94.0%, 98.3%, and 99.4% of the total variance becomes explainable, respectively. The score plot (D) distinguished extracts from northern (2L/Fl, 2Gj/SL), (5-L/Fl, 5-Gj/SL) and southern areas (3-L/Fl, 3-Gj/SL), (4-L/Fl, 4-Gj/SL) that were able to discriminate the central region (1-L/Fl, 1-Gj/SL) on the plane for both the leaf and flowermaterial samples. Changes in orientations of these three regional samples on the plane may also be associated with the presence of electroactive ingredients (e.g., polyphenols, flavonoids, other aromatics, natural pigments, and ions) subjected to anodic redox reactions at a detection potential of +750 mV (vs. Ag/AgCl) on a glassy carbon working electrode in constant potential chronoamperometry mode. Thus, the reason the samples are located closer to each other on the plane may be related to their ingredients, which may be linked to locational altitudes and climate conditions. The outcomes of the Cluster Analysis are summarized ina dendrogram (E), where it is clearly distinguished that five main clusters are using Euclidean distances. These clusters are formed by 2-(SL), 5-(SL), 2-(Fl), 5-(Fl) (red); 3-(SL), 4-(Fl) (green); 1-(Fl), 3-(Fl) (gray), 1-(SL) (blue); and 4-(SL) (violet). Even though there are five clusters in the dendrogram (E), their locations on the plane are similarly related to the PCA score plot (D).

Chemometric analysis and chemoinformatics have not been extensively studied concerning phenolic compounds isolated from *Hypericum perforatum* based on raw PDA and ECD data. To the best of our knowledge, this study represents the first time such an approach has been achieved. Thus, at this time, we cannotcompare our results with the literature; nevertheless, we may still compare our findings with other studies dealing with chemoinformatics on *Hypericum* sp. from different locations or regions of the world. Dresler and colleagues (2018) [22] performed a Principal Component Analysis (PCA) on the secondary metabolites in the leaves and stems of *Hypericum* sp. in Slovakia and Poland, successfully discriminating between samples based on their antioxidant phytochemical contents.

Unlike our approach, Dresler and colleagues did not use raw HPLC data; instead, they focused on the flavonoid content. Seyis and colleagues (2020) [24] attempted to use PCA and CA based on the determined content of polar and volatile phytochemicals to differentiate *Hypericum perforatum* populations from various locations and altitudes in Türkiye. Cirak and colleagues (2017) [26] performed PCA and CA on extracts of *H. polyphyllum* and *H. androsaemum*, successfully discriminating them based on their chemical composition according to different altitudes. Again, this differs from our approach, as they performed chemometrics using the determined phytochemical contents rather than the HPLC raw data. Overall, the chemometric approach demonstrates the utility of raw chromatographic data in distinguishing samples by plant part and geographic origin, even when traditional compound-based comparisons may be limited.

## 3. Materials and Methods

### 3.1. Standards and Reagents

The polyphenol and flavonoid standards gallic acid monohydrate (≥99%), (+)-catechin (≥99.0%), (−)-epicatechin (≥98.0%), hyperoside, quercetin (≥95.0%), and hyperforin (≥85%) were purchased from Sigma-Aldrich(Burlington, Massachusetts, USA)with the following catalog numbers:27645-250G-R, 43412-10MG, E4018-5MG, 00180585-25MG, Q4951-10G, and H5160-250UG, respectively. Gradient-grade acetonitrile (34851-2.5L) (mobile phase B) was purchased from Sigma-Aldrich. Freshly produced ultrapure water was used for all aqueous purposes (TOC < 10 ppb and total ion resistance> 18.2 MΩ/cm). The membranes used for sample filtration (0.22 or 0.45 µm) were obtained from Sartorius AG (Minisart, 16532 and 16537, respectively; Göttingen, Germany). Phosphoric acid, H_3_PO_4_ (HPLC grade), was supplied by Sigma-Aldrich (04107-2.5L), and its 0.01 M aqueous solution was used as mobile phase A.

### 3.2. Instrumentation

For routine laboratory operations, a NUVE MK 418 magnetic stirrer with an integrated heater (600 W, 1200 rpm) and a Bandelin Sonorex RK 52 ultrasonic bath (240 W, 35 kHz, with a heater) were used. An analytical balance from A&D (HR120, readability: 0.1 mg; capacity: 120 g) was used for precise and accurate weighing. Ultrapure water was obtained using three purification systems: ELGA Classic UV MK2 (Type I, 18.2 MΩ·cm), ELGA Purelab Prima (reverse osmosis, feed-grade), and Millipore Elix Milli-Q Gradient A10 (Type I, TOC < 5 ppb, resistivity ≥ 18.0 MΩ·cm). The Thermo Scientific DionexUltiMate 3000 HPLC system comprised a gradient pump (LPG-3400SD with integrated degasser), solvent rack (SR-3000), autosampler (WPS-3000TSL), column compartment (TCC-3000SD), and PDA detector (DAD-3000). Chromeleon Client (version 6.80 SR13, Thermo Scientific, Waltham, MA, USA) was used for chromatographic data acquisition, integration, and post-run analysis.The analytical column was a Thermo Scientific Syncronis C18 (250 mm×4.6 mm, ODS 100 Å, 5 µm) without a guard.

### 3.3. Plant Material

The St. John’s wort plant material was collected in five localities at different altitudes and climates in the Mediterranean and Alpine biogeographic regions [28]. The plant material was collected in 2022 in the flowering phase, which corresponded with the first and second weeks of June at the lower altitudes of the region, up to 800 m, such as the Kruja, Librazhdi, and Gjirokastraareas, and at the end of June and during the first week of July in the higher altitudes of the Alpine biogeographic region, such as those in the Pogradeci and Kukësiareas (Figure 6A and Figure 7A). The collected fresh samples were divided into flowers, leaves, and stems. A quantity of 2 kg, consisting of flowers and stems with leaves, was dried in the dark at room temperature, 35–40 °C. The voucher specimens from each locality were deposited at the Department of Biology and in the National Herbarium of the University of Tirana.

### 3.4. Extraction of Plant Material

Prior to the drying stage, the medicinal plant material was segregated based on anatomical parts. Floral tissues were processed independently, while stems and leaves were combined. After the drying process on the shadow, the plant material was finely ground by a mill (Brabender OHG, Duisburg). The plant material extraction process was performed using a modified method described by Wagner and Bladt (1996) [21]. Methanol at 80 °C was used for Soxhlet extraction. The extracts were dried under a vacuum. After the extract lyophilization (Labconcolyophilizer, −50 °C; Kansas City, MO, USA), a portion was weighed and dissolved in 4 mL of HPLC-grade methanol. The dissolved extracts were then filtered through 0.45 μm pore size membrane filters (Sartorius AG; 16555 Minisart) and passed into a vial before being injected into the HPLC system.

### 3.5. Mobile Phase Preparation

For both the PDA and ECD detectors, HPLC separation was performed using aqueous 0.01 M H_3_PO_4_ as mobile phase A and gradient-grade (≥99.9%) acetonitrile as mobile phase B. Mobile phase A was freshly prepared by boiling and cooling ultrapure water, and then filtered through a 0.22 μm membrane filter before use.

### 3.6. Standard and Sample Solution Preparations

Working standard solutions were prepared at three successive concentrations: 2.5, 5.0, and 10.0 ppm.Multi-injections (n = 3) were performed for each level to comply with the method validation guideline ICH Q2(R1). Related calibration graphs were obtained using both PDA and ECD detectors and utilized to quantify the methanolic extracts of the plant samples. Precisely weighed extracts were dissolved in methanol to prepare stock solutions at a concentration of 1000 ppm. Achieving the necessary precision and accuracy is essential; therefore, calibrated autopipettes with the appropriate volume capacity were employed. Standards and sample stability were ensured by quickly pipetting solutions into amber flacons and storing them at 5 °C. Due to the fact that quercetin and hyperforin undergo oxidation in solution, all standard and sample solutions were freshly prepared, stored in amber vials, and kept at 5 °C. The maximum shelf life of these solutions is limited to 48 h, and all injections were performed within this time frame to ensure analytical integrity

### 3.7. HPLC-PDA and HPLC-ECD Analysis

The gradient program for PDA and ECD elution was optimized as follows: 0–3 min: A (95%), 3–10 min: A (55%), 10–20 min: A (100%), and 20–25 min: A (5%), 25–30 min: A (5%). The thermostated column compartment maintained the analytical column at 25 °C (ECD) or 45 °C (PDA), while injection volumes were 3 μL for standards and 5 μL for sample extracts, respectively. The analytical column was a Hypersil C18 column with dimensions of 250 mm × 4.6 mm, a particle size of 5 µm, and a pore size of 100 Å. A flow rate of 0.25 mL/min was employed, and each compound was tentatively identified by its unique retention time. In the case of PDA, UV-Vis 3D spectra were also used for discrimination and identification under the same conditions. Quantitative determinations were performed using external standard addition methods based on peak height or area. PDA acquired UV-Vis spectra in the 300–700 nm range, and absorbance was sampled in four simultaneous channels at 230, 270, 367, and 590 nm wavelengths. Regarding ECD, electrochemical detection was based on constant potential chronoamperometry performed at +750 mV (vs. Ag/AgCl) on the surface of the glassy carbon working electrode.

### 3.8. Method Validation (HPLC-PDA)

The HPLC-PDA method was validated according to ICH Q2 (R1), assessing specificity, linearity (1–10 ppm, R^2^ > 0.996), accuracy (recoveries at 2.5–10 ppm), precision (RSD < 5%), and sensitivity (LOD/LOQ: 0.24–0.61 µg/mL). No matrix interferences were observed.

Overall, the method demonstrated strong analytical performance and is suitable for quantitative profiling of phenolic constituents in Hypericum perforatum extracts.

### 3.9. Method Validation (HPLC-ECD)

The HPLC-ECD method was similarly validated, showing strong linearity (R^2^ > 0.999), acceptable recoveries, precision (RSD < 5%), and comparable sensitivity to PDA. Electroactive compounds such as gallic acid and quercetin were selectively detected.

The HPLC-ECD method complements PDA detection by enhancing selectivity for phenolic compounds with electrochemical activity, offering robust quantification in complex plant matrices.

### 3.10. Chemometric Analysis

The statistical software used for cheminformatics was Minitab (v.17.1.0). Briefly, the experimental raw data wasinitially converted to txt formatand imported from the corresponding data processing software to an MS Excel worksheet. The experimental data was stored in separate Excel files for each hardware or method, subsequently imported into Minitab software, and then edited using the data reduction process, which involved subtracting the mean from each data value and then dividing the result by the standard deviation. Consequently, columns of reduced data were transposed merely by converting them into rows. Finally, the reduced and transposed data worksheet was analyzed using Principal Component Analysis (PCA) and Cluster Analysis (CA). The PCA was performed using the “Principal Components” in the start menu and the multivariate sub-menu of the software. The PCA provided scree plots, score plots, and loading plots for the desired principal components. The CA was carried out using the “cluster observations” comments in the start menu and multivariate sub-menu of the software. As a result of the CA, a dendrogram plot was generated using Euclidean distances to classify observations (raw data) based on their similarity

## 4. Conclusions

This study presents the first comprehensive HPLC-PDA/ECD profiling and chemometric analysis of *Hypericum perforatum* L. from Albania. By integrating dual-mode detection with Principal Component Analysis and Cluster Analysis, we achieved effective discrimination of plant materials by geographic origin and tissue type. The method demonstrated high sensitivity, reduced elution time, and enabled the use of raw chromatographic data for sample classification—an advancement over traditional compound-based approaches. These findings contribute to the understanding of climate-linked phytochemical variability and support the development of quality standards and traceability frameworks for medicinal plant resources in Southern Europe.

## Figures and Tables

**Table 1:** Flavonoid contents of *H. perforatum* methanol extracts based on PDA and ECD detection (Mean ± St. Dev; n = 3 injections per sample).

HPLC-PDA							
Sample	Gallic acid (ppm)	Catechin (ppm)	Epicatechin (ppm)	Hyperoside (ppm)	Quercetin (ppm)	Hyperforin (ppm)	Total (ppm)
1-Gj/SL	4.77 ± 0.19	*ND	0.31 ± 0.01	53.32 ± 2.13	0.89 ± 0.04	53.38 ± 2.14	112.67 ± 4.51
2-Gj/SL	4.11 ± 0.16	ND	0.29 ± 0.01	76.23 ± 3.05	1.41 ± 0.06	50.74 ± 2.03	132.78 ± 5.31
3-Gj/SL	5.24 ± 0.21	ND	ND	71.38 ± 2.86	1.46 ± 0.06	114.47 ± 4.58	192.55 ± 7.7
4-Gj/SL	6.20 ± 0.25	ND	ND	52.47 ± 2.10	0.89 ± 0.04	41.70 ± 1.67	101.26 ± 4.05
5-Gj/SL	4.92 ± 0.20	ND	ND	96.12 ± 3.84	0.78 ± 0.03	40.96 ± 1.64	142.78 ± 5.71
1-L/Fl	4.80 ± 0.19	ND	0.30 ± 0.01	27.09 ± 1.08	0.84 ± 0.03	69.26 ± 2.77	102.29 ± 4.09
2-L/Fl	4.34 ± 0.17	ND	ND	62.73 ± 2.51	1.26 ± 0.05	79.87 ± 3.19	148.2 ± 5.93
3-L/Fl	4.65 ± 0.19	ND	ND	52.31 ± 2.09	1.63 ± 0.07	142.64 ± 5.71	201.23 ± 8.05
4-L/Fl	6.05 ± 0.24	ND	0.31 ± 0.01	50.92 ± 2.04	1.11 ± 0.04	70.43 ± 2.82	128.82 ± 5.15
5-L/Fl	5.87 ± 0.23	ND	0.52 ± 0.02	85.36 ± 3.41	1.14 ± 0.05	89.54 ± 3.58	182.43 ± 7.3
**HPLC-ECD**							
**Sample**	**Gallic acid (ppm)**	**Catechin (ppm)**	**Epicatechin (ppm)**	**Hyperoside (ppm)**	**Quercetin (ppm)**	**Hyperforin (ppm)**	**Total (ppm)**
1-Gj/SL	4.40 ± 0.18	*ND	0.59 ± 0.02	57.42 ± 2.3	0.49 ± 0.02	47.25 ± 1.89	110.15 ± 4.41
2-Gj/SL	3.75 ± 0.15	ND	0.76 ± 0.03	78.92 ± 3.16	1.69 ± 0.07	45.52 ± 1.82	130.64 ± 5.23
3-Gj/SL	5.70 ± 0.23	ND	0.41 ± 0.02	73.72 ± 2.95	1.33 ± 0.05	101.37 ± 4.05	182.53 ± 7.30
4-Gj/SL	7.15 ± 0.29	ND	0.23 ± 0.01	49.39 ± 1.98	0.91 ± 0.04	41.43 ± 1.66	99.11 ± 3.96
5-Gj/SL	4.75 ± 0.19	ND	0.90 ± 0.04	101.48 ± 4.06	0.85 ± 0.03	38.47 ± 1.54	146.54 ± 5.86
1-L/Fl	4.70 ± 0.19	ND	0.21 ± 0.01	25.53 ± 1.02	0.96 ± 0.04	61.39 ± 2.46	92.79 ± 3.71
2-L/Fl	4.50 ± 0.18	ND	0.72 ± 0.03	69.55 ± 2.78	1.89 ± 0.08	70.41 ± 2.82	147.07 ± 5.88
3-L/Fl	4.75 ± 0.19	ND	0.38 ± 0.02	50.52 ± 2.02	1.47 ± 0.06	128.4 ± 5.14	185.52 ± 7.42
4-L/Fl	5.30 ± 0.21	ND	0.59 ± 0.02	49.34 ± 1.97	1.38 ± 0.06	66.29 ± 2.65	122.90 ± 4.92
5-L/Fl	4.65 ± 0.19	ND	0.83 ± 0.03	88.83 ± 3.55	1.45 ± 0.06	91.37 ± 3.65	187.13 ± 7.49

Note: Values are reported with standardized significant digits based on analytical precision. Relative standard deviation (RSD) and 95% confidence intervals were calculated for each compound. Abbreviation: * ND: not detected; Gj/SL:stem and leaf; and L/Fl:flower.

## Data Availability

The data supporting the reported results can be found in the text.
